# Constructing an Individualized Middle Cerebral Artery Model Using 3D Printing and Hydrogel for Bypass Training

**DOI:** 10.7759/cureus.16749

**Published:** 2021-07-30

**Authors:** Sinem S Ovunc, Mohamed Yassin, Ricky Chae, Adib Abla, Roberto Rodriguez Rubio

**Affiliations:** 1 Neurological Surgery, University of California San Francisco (UCSF), San Francisco, USA

**Keywords:** immersive technologies, simulation, three-dimensional (3d) printing, self-paced learning, medical education, surgical training

## Abstract

The importance and complexity of cerebral bypass surgery (CBS) highlight the necessity for intense and dedicated training. Several available training models are yet to satisfy this need. In this technical note, we share the steps to construct a digital imaging and communications in medicine (DICOM)-based middle cerebral artery (MCA) model that is anatomically accurate, resembles handling properties of living tissue, and enables trainers to observe the cerebrovascular anatomy, improve and maintain microsurgical dexterity, and train in the essential steps of CBS.

The internal and external molds were created from the geometry of DICOM-based MCA using Fusion 360 software (Autodesk, San Rafael, USA). They were then three-dimension (3D) printed using a polylactic acid filament. The 15% w/v solution of polyvinyl alcohol (PVA) was prepared and injected between the molds. Using five freeze-thaw cycles the solution was converted to tissue-mimicking cryo-gel. The model was then placed in a chloroform bath until the internal mold dissolved. To evaluate the accuracy of the MCA model, selected characteristics were measured and compared with the MCA mesh.

The DICOM-based MCA model was produced using 3D printing that was available in the lab and the overall cost was less than $5 per model. The external mold required six and a half hours to be 3D printed, while the internal mold only required 23 minutes. Overall, the time required to 3D print the DICOM-based MCA model was just short of seven hours. The greatest statistically significant difference between the virtual MCA model and the DICOM-based MCA model was found in the length of the pre-bifurcation part of the M1 segment and the total length of the superior bifurcation trunk of M1 and superior branch of M2. The smallest statistically significant difference was found at the diameter of the inferior post-bifurcation trunk of the M1 segment and the diameter at the origin of the artery.

This technical report aims to show the construction of a CBS training system involving the DICOM-based MCA model that demonstrates the shape of the vascular tree, resembles the handling/suturing properties of living tissue, and helps set up a homemade training station. We believe that our DICOM-based MCA model can serve as a valuable resource for CBS training throughout the world due to its cost-effectiveness and straightforward construction steps. Moreover, once the DICOM-based MCA model is used with our training station, it may offer an option for trainers to gain and maintain CBS skills despite limitations on time, cost, and space.

This work was presented in February 2019 at the American Association of Neurological Surgeons/Congress of Neurological Surgeons (AANS/CNS) Cerebrovascular Section Annual Meeting held in Honolulu, Hawaii.

## Introduction

In neurosurgery, there are many procedures that a skilled neurosurgeon must master. One such procedure is the cerebral bypass. Any brain surgeon dealing with cerebral vasculature requires precise actions while navigating the blood vessels on the exterior surface of the temporal lobe [1], microanastomosis skills, familiarity with the shape and volume of the vessels, and the ability to use instruments properly for basic vessel repairment or complex revascularization procedures [2]. A recent study has demonstrated that the number of cerebral bypasses performed for adult revascularization has increased in the United States between the years 2002 and 2014. However, its application for the treatment of occlusive vascular disease and cerebral aneurysms has decreased [3].

This procedure comes with its challenges and a neurosurgeon must be highly trained in augmentation and restoration to overcome them. Training will not only help in reducing complications but will also add confidence to the neurosurgeon as they perform a high-risk procedure [2]. The importance and complexity of the cerebral bypass highlight the necessity of training to improve and maintain microanastomosis skills. Efficient training must simulate the real operative conditions using accessible, inexpensive, reusable, and anatomically correct models that provide haptic feedback. Many resources for training outside the operating room exist, including animal models, such as turkey/chicken brachial arteries [4]. Each model has its own strengths and weakness.

In the initial stages of practicing cerebrovascular bypass surgery (CBS) skills, latex gloves or silastic tubes can be useful models that demonstrate high construct validity. However, they do not provide realistic handling properties of cerebral tissue since multiple factors, such as localized vessels, will affect this procedure [5]. On the other hand, live animal models are close to real surgery conditions because of their natural blood flow, the potential for thrombosis, and similarity to human tissue. Even so, ethical regulations on living animal models render this option unfeasible. Additionally, live animal models require vivarium and animal care staff, several pharmaceuticals, and knowledge of anesthesia for animals. Turkey and chicken wing/leg could be better options since they still have tactile feedback and are not restricted by ethical regulations and institutional review board approval [2]. Human cadavers provide anatomical certainty but are susceptible to infections and require dedicated laboratories. Moreover, fixation solutions make the vessel wall tissue stiff and friable.

New training models have emerged in several fields of medicine with recent advances in three-dimensional (3D) printing and virtual reality technologies. Especially in the field of neurosurgery, creating 3D models for surgical training and preoperative planning is of paramount importance due to the complexity of procedures and insufficiency of 2D images for demonstrating spatial relationships between nerves, arteries, veins, brain, and skull [6]. Even as 3D printing and virtual reality become more relevant, it is important to note that they are unable to provide physical hands-on experience that is crucial for all neurosurgeons-in-training.

Recently, 3D printing evolved with the use of biological materials as the substrate. This concept, known as 3D bioprinting, has made its presence in regenerative medicine, tissue engineering, drug and cancer research, and organ transplantation. Various studies have revealed that bioprinting mimics native vascular networks for tissue engineering in the growth of thick tissue [7]. Although bioprinting has strong potential for constructing vessels, limitations associated with cost and infrequent applications do not make it a preferred method for constructing models for CBS training and preoperative planning [8].

Despite significant improvements in current technologies, constructing vessels with high accuracy using 3D printing remains difficult due to the hollow shape of vessels, their curvature, small size, and special characteristics of wall tissue. Furthermore, even if 3D printers can facilitate the construction of anatomically correct models, there are not many satisfactory printing materials that mimic the handling properties of vessel wall tissue. To make up for what 3D printing lacks, we aimed to integrate our model with polyvinyl alcohol cryogel (PVA-C). PVA-C was chosen because it has human tissue-mimicking properties and can be easily manipulated due to its high elasticity [9].

Depending on the required treatment, various types of cerebral bypass procedures are performed on different arteries. In this study, we chose to construct models of the middle cerebral artery (MCA) due to its frequent applications in CBS training [2].

We highlight the workflow for constructing MCA models through 3D printing and PVA-C to generate models that are anatomically accurate, cost-effective, easily constructible, and resemble handling properties of living tissue, and aids in the training of CBS skills. Our workflow includes generating molds from a virtual model of MCA using computer-aided design tools and 3D printing, preparing and injecting polyvinyl alcohol (PVA) solution as a tissue-mimicking material, applying freeze/thaw cycles to convert the PVA solution to PVA-C, discarding the external mold, and dissolving the internal mold using chloroform. We also developed a CBS training station using a tripod and smartphone camera to further demonstrate how our MCA models can be utilized without expensive surgical equipment and tools.

## Technical report

Material and methods

Fabrication of the Internal Mold

**Figure 1:**
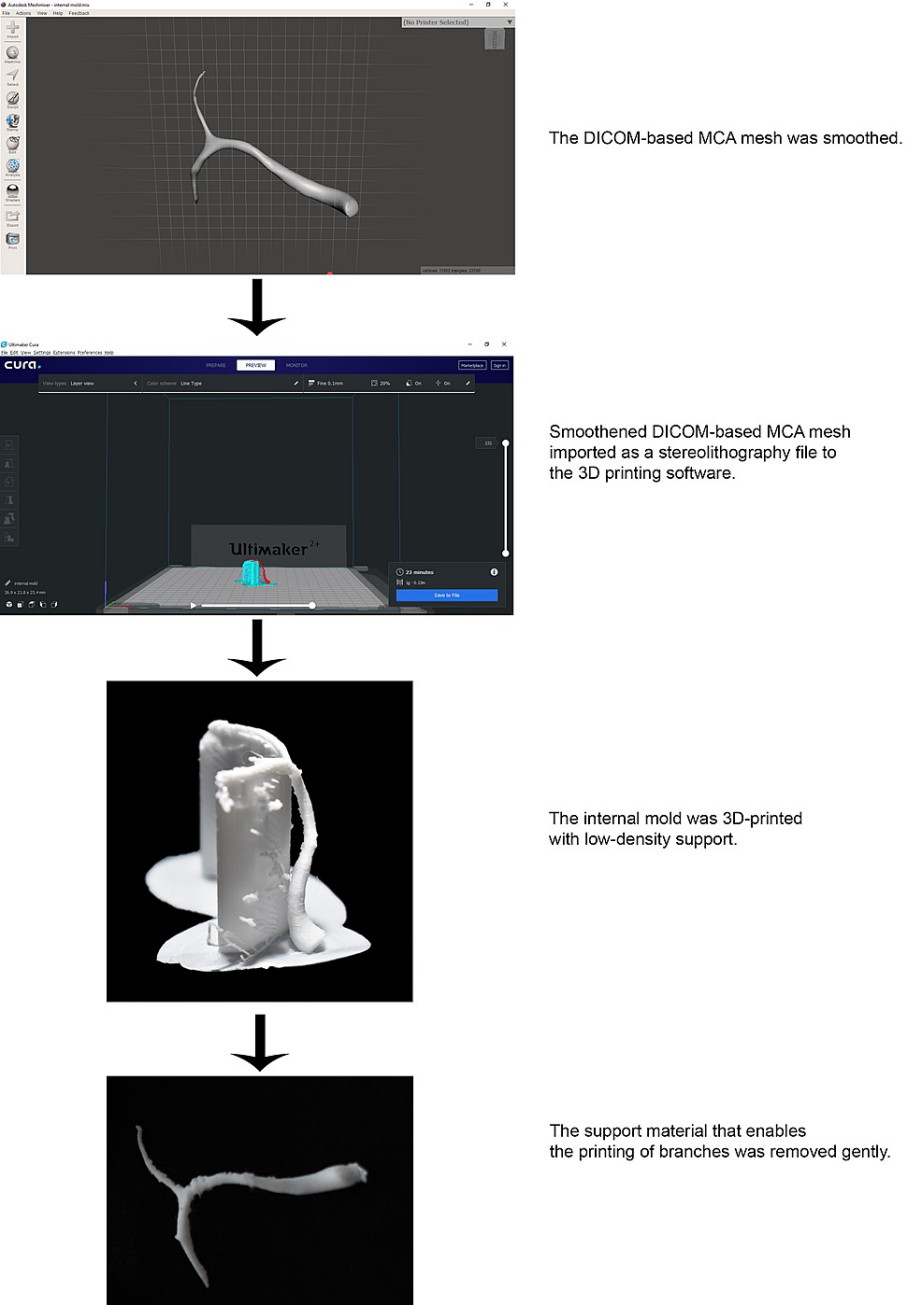
Fabricating the internal mold via 3D printing

The virtual model of MCA can be generated by extracting data from digital imaging and communications in medicine (DICOM) and images of computed tomographic angiography (CTA). We downloaded a DICOM-based virtual model of MCA from an online resource (thingiverse.com) and smoothed it using computer-aided design (CAD) software namely Meshmixer (Autodesk, San Rafael, USA), to decrease the volume of the model without changing its shape and angles. The resulting model, which represents the virtual model of the internal mold, was exported as a stereolithography (STL) file and uploaded to Ultimaker Cura (Ultimaker B.V, Geldermalsen, Netherlands), an open-access 3D printing software. The virtual model was then rotated until its branches were placed on top and the opposite part was placed below to print anatomically accurate branches. Brim, which adds a single layer flat area around the base of the model to discourage warping, was chosen from the build plate adhesion settings on the 3D printing software. Following that, low-density supports that prevent the overhanging parts of the model from falling were generated. These supports were removed post-printing. Polylactic acid (PLA) thermoplastic was selected as the 3D printing material due to its high solubility in chloroform that facilitates the removal of the internal mold. Finally, the virtual model of the internal mold was 3D-printed on Ultimaker 2+ (Ultimaker B.V, Geldermalsen, Netherlands) (Figure 1). Due to the fragile character of the internal mold, the support material that enables the printing of branches was removed gently.

Design and Fabrication of the External Mold

**Figure 2:**
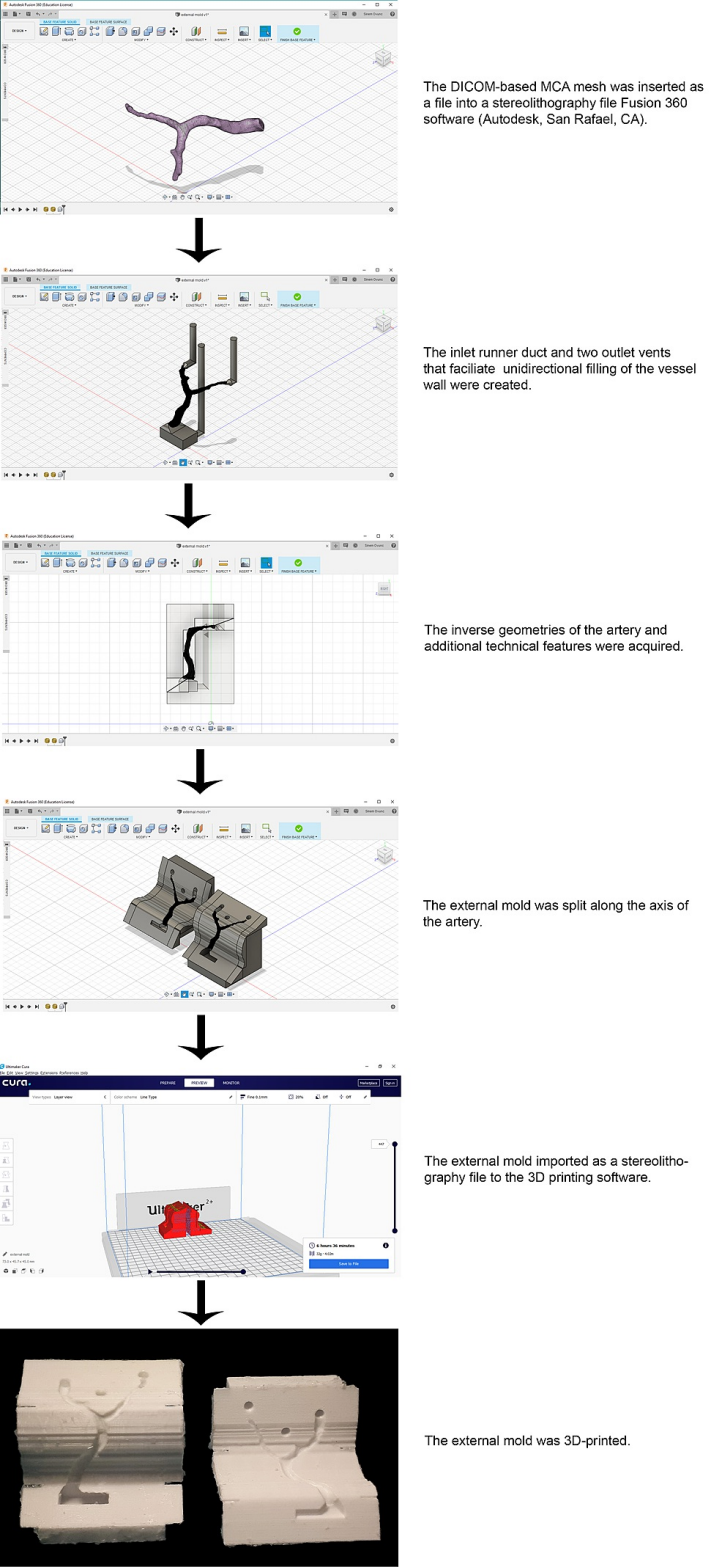
Fabricating the external mold via 3D printing

The DICOM-based virtual model of MCA was imported as an STL file into the CAD software Fusion 360 (Autodesk, San Rafael, USA). Create Base Feature was selected to enter direct editing mode. In this mode, the STL file appears in mesh form. This mode also allows users to create objects in another form named solid body. As the mesh form of the DICOM-based virtual model could not be used as reference geometry for the external mold, it was converted to a solid body by using the Mesh to B-Rep conversion tool and then connected with boxes by the loft tool to create a shape that transitions between two or more edges or faces. Then came the sketches. 2D geometries that use a base to construct 3D geometries were created and used as paths to add three solid pipes (an inlet runner duct and two outlet vents) that facilitate unidirectional filling of the vessel wall. A box was created according to the earlier designed parts of the external mold to be the target body. The parts that would be hollow in the completed external mold were then created. These parts were then excluded from the box that would be the body of the external mold. The workspace was switched to the patch environment to create and extrude a fit point spline along an axis of arteries. It was used as a tool plane to split the external mold for easy removal of the DICOM-based MCA model with the internal mold inside. Small boxes were added to two sides of the external mold to enable easy handling. The completed external mold was exported and converted to STL file format. It was then 3D printed via Ultimaker 2+ (Ultimaker B.V, Geldermalsen, The Netherlands) (Figure 2).

Preparation of PVA Solution

To prepare a 15% w/v aqueous solution of PVA, 5g of PVA powder was added to 23.5ml of deionized water. The solution was heated and stirred at 100°C for approximately one hour until it became clear. The solution was left to cool to room temperature for approximately one hour. Finally, 9.8ml of dimethyl sulfoxide (DMSO) was added to the solution. Throughout the preparation process, the solution was covered with stretch film to prevent drying.

Injection of PVA Solution

The internal mold was mounted between two parts of the external mold and assembled. The constructed double-layered mold was sealed with silicone to prevent leaking during freeze/thaw cycles. The PVA solution was injected into the inlet runner duct of the mold using a 1ml syringe until the overflow at the outlet vents was seen. Overall, 3ml of PVA solution was used per model.

The Freezing and Thawing Process

Multiple freezing and thawing cycles were performed to convert the PVA solution into a gel. This allowed the material to form a porous network of hydrogen bonds and simulate characteristics of the artery wall.

The mechanical properties of the material were improved by altering the concentration of the solution and the number of freeze-thaw cycles based on feedback received from surgical experiences. Five freeze-thaw cycles were subsequently administered to the entire external mold. Each cycle consists of two steps. Step one involves placing the mold in a −20 °C freezer for 24 hours. And step two is the thawing of the mold at 4 °C for 24 hours.

Removal of the Internal Mold

**Figure 3:**
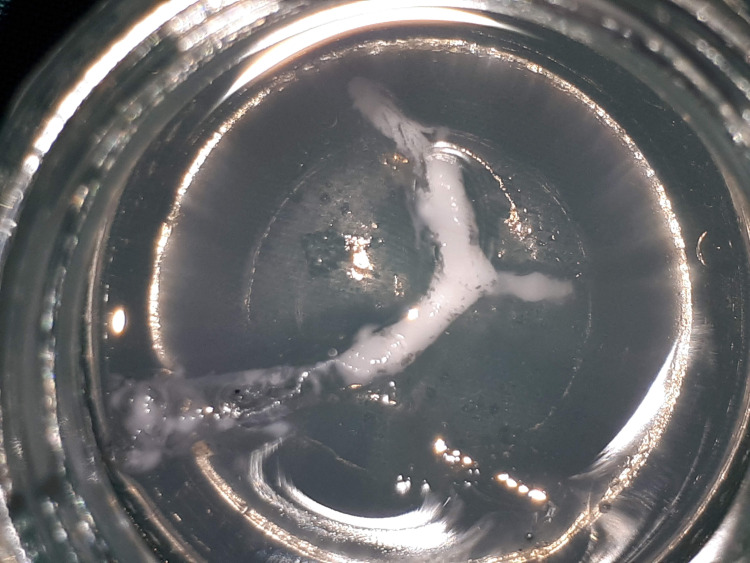
The model was placed in the chloroform bath until the internal mold dissolved

The two parts of the external mold were separated and the model with the internal mold was taken out. The internal mold-model complex was placed in a chloroform bath. After 24 hours, the internal mold dissolved completely without impacting the physical model (Figure 3).

Training Station

**Figure 4:**
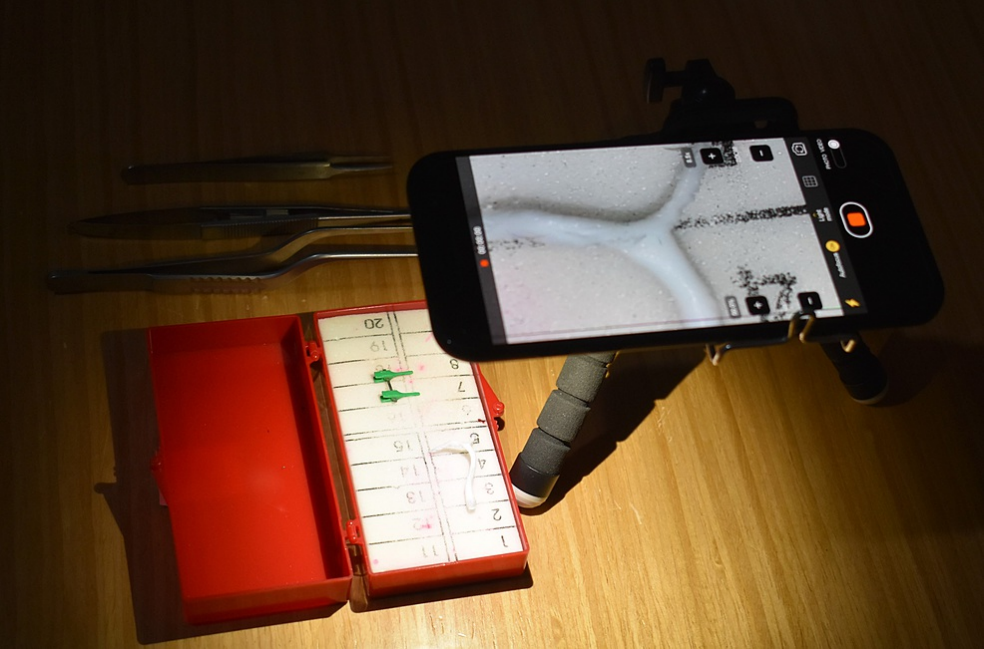
The training station was constructed using a tripod and a smartphone

A training station was constructed using a smartphone and a tripod. The camera application on the smartphone was used to replicate the magnified view provided by the surgical microscope, and the tripod was used to stabilize the smartphone to arrive at the desired position (Figure 4).

Statistical Analysis

**Figure 5:**
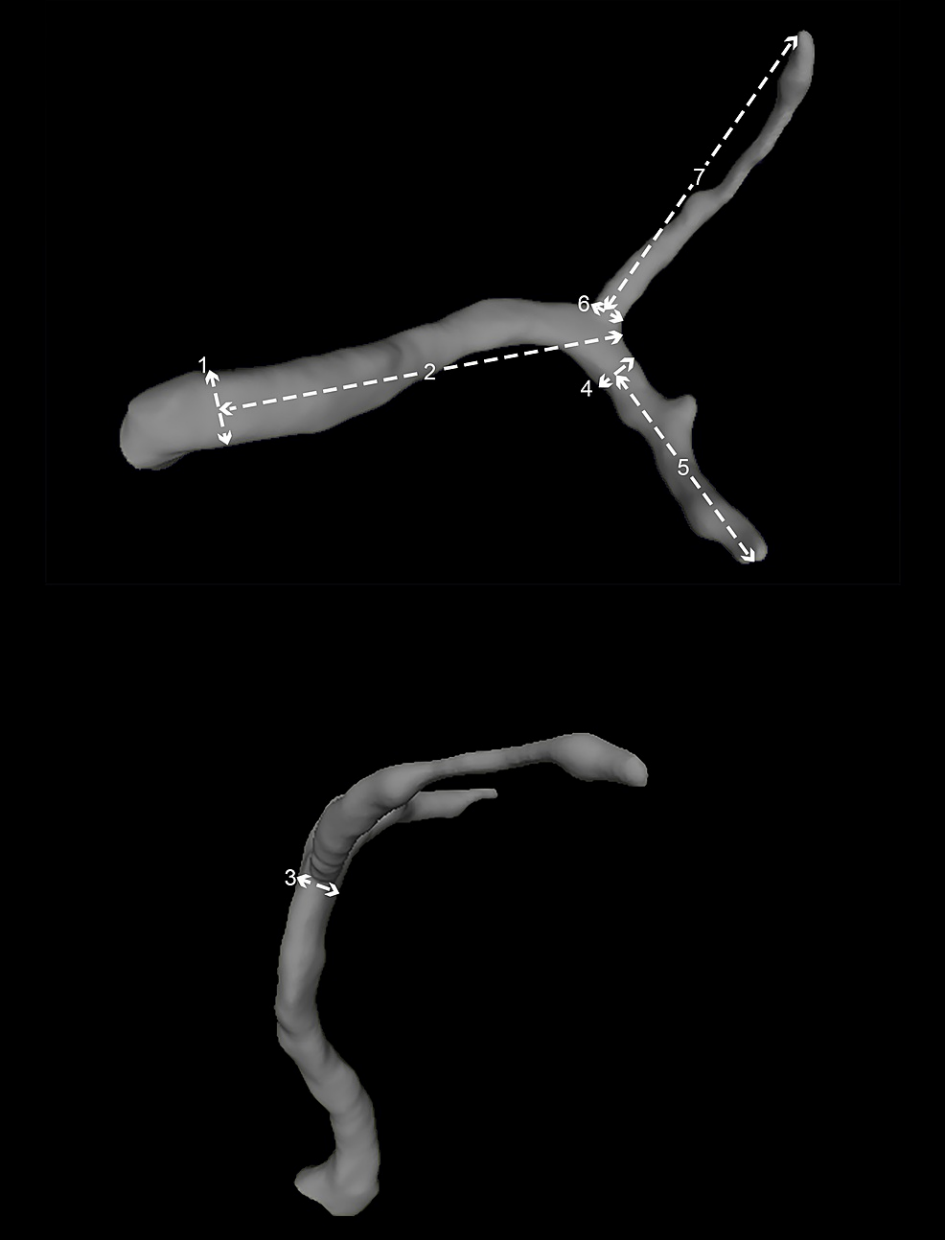
Depiction of the anatomical characteristics of  MCA that were measured via MeshLab software (ISTI-CNR, Pisa, Italy) for the mesh and a digital caliper for the model

We evaluated the accuracy of our protocol by comparing measurements between the DICOM-based virtual model of MCA numbering one (n=1) and the mean of the DICOM-based MCA models (n=8). The following anatomical characteristics of MCA were measured using MeshLab software (ISTI-CNR, Pisa, Italy) for the DICOM-based virtual model of MCA and a digital caliper for the DICOM-based MCA models: (1) diameter of the artery at its origin, (2) length of the pre-bifurcation part of the M1 segment, (3) diameter of the artery at the bifurcation point, (4) diameter of the inferior post-bifurcation trunk of the M1 segment, (5) total length of the inferior post-bifurcation trunk of M1 and inferior branch of M2, (6) diameter of the superior post-bifurcation trunk of the M1 segment, (7) total length of the superior post-bifurcation trunk of M1 and superior branch of M2. These measurements can be seen in Figure 5. 

The anatomical characteristics were adopted from the literature [10]. The MCA consists of four segments, M1 through M4. The M1 segment begins at the origin of the MCA, where the internal carotid artery divides and gives rise to the MCA and anterior cerebral artery, and terminates at the limen of the insulae. The M2 segment begins at the limen of the insulae and terminates at the circular sulcus of the insulae. The M1 segment may bifurcate or trifurcate before forming the M2 segment. In our DICOM-based virtual model of MCA, a bifurcation was examined. We divided our DICOM-based MCA model into two parts according to the bifurcation of the M1 segment for the measurements.

The mean and standard errors were reported for each measured parameter. One-sample t-tests were performed to assess any statistically significant differences between measurements of the DICOM-based virtual model of MCA and DICOM-based MCA models. All statistical analyses were performed using John's Macintosh Project (JMP), version 14 (SAS, Cary, NC, USA).

Results

Suturable, hollow, DICOM-based MCA model was acquired with the above-proposed method. The filament cost was less than $1 for two pieces of the external mold. Compared to the external mold that required 32g of filament and six and a half hours for 3D printing, the internal mold required only 1g of filament and 23 minutes for 3D printing. A PVA solution of 33.3ml was prepared using 5g PVA powder, 23.5ml deionized water, and 9.8ml DMSO. However, only 3ml of the PVA solution was spent per DICOM-based MCA model. The average costs were $15 per 100g of PVA powder, $15 per liter of deionized water, and $20 per 236.6ml (8oz) of DMSO. The total material cost per DICOM-based MCA model was less than $5. We relied on in-house 3D printing services provided by our institution. As for the training station, the average cost of the tripod was $2. The cost of the smartphone wasn’t included as it is a widely available personal device. With the addition of surgical instruments (needle holder, forceps, micro scissor, and 10-0 suture) the total cost of constructing our CBS training system is $129 (Table 1). 

**Table 1:** List of costs incurred in constructing the DICOM-based MCA model that includes the training station, materials used, and microsurgical instruments and equipment. Cost of smartphone and 3D printer were excluded as the former is a personal device and for the latter, we relied on the one provided by our institution.

Software and Materials	Where to Find	Average Cost	Amount per Model	Total Cost per Training Module
Training Station				
Smartphone	Personal device		1	-
Phone application DSLR Zoom Camera (Peakercorp Dev Team)	Play Store or similar apps at App Store	Free app		$0
Tripod	ebay.com	$2	1	$2
The total cost of the training station		$2		
Surgical equipment and expendables				
Microneedle holder	ebay.com, amazon.com	$12	1	$12
Tying micro forceps	ebay.com, amazon.com	$15	1	$15
Microscissor (or a normal scissor)	ebay.com, amazon.com	$10	1	$10
10-0 suture	ebay.com, amazon.com, alibaba.com	$3	1	$3
Pen	Stationery store			
The total cost of surgical equipment		$40		
Materials used in model preparation				
Polylactic acid (PLA) filament	ebay.com, amazon.com, alibaba.com	$25/kg	32+1g	$0.83
Polyvinyl alcohol (PVA) powder	ebay.com, amazon.com, alibaba.com	$15/100g	<1g	
Deionized water (low grade)	ebay.com, amazon.com, alibaba.com	$15/L	<3ml	
Dimethyl sulfoxide (DMSO)	ebay.com, amazon.com, alibaba.com	$20/ 8oz (236.6ml)	<1ml	
Silicone tube sealant	ebay.com	$5	little	
Syringe	ebay.com, amazon.com, alibaba.com	$1	1	
Veterinary needles	ebay.com, amazon.com, alibaba.com	$6/pack (12/pack)	1	
Others (oven, refrigerator, pat, glassware, bar, stretch film, etc.)	Found in every home	-	-	-
The total cost of the CBS training module (including training station, neurosurgical equipment, and materials required to produce DICOM-based MCA model)		$129		

Statistically significant differences were observed when comparing measurements of the DICOM-based virtual model of MCA to those of the mean of the DICOM-based MCA models (p<0.002). The greatest mean differences between the DICOM-based virtual model of MCA and DICOM-based MCA models were observed in the length of the pre-bifurcation part of the M1 segment (-0.300mm) and total length of the superior post-bifurcation trunk of M1 and superior branch of M2 (-0.275mm). The smallest differences were observed in the diameter of the inferior post-bifurcation trunk of the M1 segment (-0.075mm) and the diameter of the artery at the origin (-0.100mm). Moreover, the greatest standard deviation (SD) of means was observed for the total length of the superior post-bifurcation trunk of M1 and a superior branch of M2 (0.128). And the smallest SD of means was observed in the diameter of the inferior post-bifurcation trunk of the M1 segment (0.071) (Table 2).

**Table 2:** Anatomical characteristics of the MCA were measured on the DICOM-based virtual model and DICOM-based MCA models. Paired t-tests were performed to assess any statistically significant differences between these models.

Landmark	DICOM-based virtual model of MCA	DICOM-based model of MCA (1)	DICOM-based model of MCA (2)	DICOM-based model of MCA (3)	DICOM-based model of MCA (4)	DICOM-based model of MCA (5)	DICOM-based model of MCA (6)	DICOM-based model of MCA (7)	DICOM-based model of MCA (8)	Mean	Standard Deviation	Mean Difference
Diameter of the artery at the origin	3.2mm	3.2mm	3mm	3.1mm	3.2mm	3mm	3.1mm	3mm	3.2mm	3.1mm	0.092582	-0.1
Length of the pre-bifurcation part of the M1 segment	21.9mm	21.7mm	21.5mm	21.5mm	21.7mm	21.5mm	21.6mm	21.6mm	21.7mm	21.6mm	0.092582	-0.3
Diameter of the artery at the bifurcation point	2.5mm	2.4mm	2.4mm	2.3mm	2.4mm	2.3mm	2.2mm	2.2mm	2.4mm	2.325mm	0.0886405	-0.175
Diameter of the inferior post-bifurcation trunk of the M1 segment	2.1mm	2.1mm	2mm	2mm	2.1mm	2mm	1.9mm	2mm	2.1mm	2.025mm	0.0707107	-0.075
Total length of the inferior post-bifurcation trunk of M1 and inferior branch of M2	12.6mm	12.5mm	12.4mm	12.5mm	12.6mm	12.3mm	12.4mm	12.4mm	12.5mm	12.45mm	0.092582	-0.15
Diameter of the superior post-bifurcation trunk of the M1 segment	1.9mm	1.8mm	1.8mm	1.7mm	1.8mm	1.7mm	1.6mm	1.7mm	1.8mm	1.7375mm	0.0744024	-0.163
Ttotal length of the superior post-bifurcation trunk of M1 and superior branch of M2	16.8mm	16.6mm	16.4mm	16.5mm	16.6mm	16.3mm	16.5mm	16.6mm	16.7mm	16.525mm	0.128174	-0.275

Once the DICOM-based MCA models were compared to the DICOM-based virtual model of MCA one-by-one, the greatest p-value was observed for the DICOM-based MCA model (4) (p=0.0453), whereas the smallest p values were observed for DICOM-based MCA model (6) and (7) (p=0.0002).

## Discussion

Cerebrovascular bypass surgery remains a complex procedure in neurosurgery. With the help of cutting-edge technology, the necessary training to maintain quality CBS skills is now easily within our reach. In this study, we constructed a DICOM-based MCA model that precisely simulates the shape of the vascular tree, mimics handling properties of living tissue, enables trainers to observe the cerebrovascular anatomy, improves and maintains microsurgical dexterity, and helps train in the essential steps of CBS. Our protocol involved creating models based on DICOM data, 3D printing, and casting.

3D printing has a wide range of applications, including implantable device and surgical tool fabrication, pharmaceutical product design, organ printing, model construction for training, and preoperative planning among many others in medicine [11]. These broad-spectrum applications are capable of providing opportunities to acquire individually designed, or DICOM-based products with various options. These options are also available in both cost-effective and time-effective ways. We benefitted from 3D printing in acquiring our external and internal molds. Molding techniques have been preferred in various studies ranging from vascular surgery [12] to neurosurgery [13] due to the need to produce soft models that have living tissue characteristics. These models enable trainers to practice surgical procedures involving clipping, cutting, suturing, stapling, and energy-based device use. Furthermore, it leads to the development of new imaging techniques and surgical approaches. In neurosurgery especially, these techniques are crucial for molding a highly skilled neurosurgeon. Different techniques and materials used for casting are selected according to the tissue and procedure involved. The material characteristic and usefulness of PVA polymer as a tissue-mimicking material has been described previously [14, 15]. We also chose to use PVA as the tissue-mimicking material.

Acquiring a hollow model is essential for vascular training or research. In the literature, many methods based on 3D printing described how to construct hollow models that replicate the complex shape of the vascular tree [12,16]. Those methods differ from each other in various ways such as choice of casting material, the number of layers on the mold, and internal mold (template) removing approach to name a few. Chee et al. established the construction of walled carotid phantom using 3D printed molds and PVA cryo-gel for vascular imaging investigations. In their study, a two-step process consists of snapping the core and retrieving the fragments to obtain the vessel model with a hollow lumen. Even if this method succeeded for their model, we found it inappropriate for our study as we aimed to construct the DICOM-based MCA model, which has complex geometry and is small compared to the CAD-based carotid model. We also preferred to use a solvent for removing the internal mold. The chloroform enables us to obtain a hollow lumen while providing minimal harm to the DICOM-based MCA model. However, our results demonstrated that there were statistically significant differences when comparing measurements of the DICOM-based virtual model of MCA to those of the mean of the DICOM-based MCA models.

According to our measurements and statistical analysis, we believe that the DICOM-based MCA model may be an efficient option for resident and medical student education, although it may be inappropriate for preoperative training.

Even if models are of paramount importance in training, the magnified view provided by a neurosurgical microscope is required for CBS training. However, conventional neurosurgical microscopes are expensive, immobile, limited in number, and not available at all times. Several systems that replicate the magnified view provided by a neurosurgical microscope were previously described to enable trainers to practice practically anywhere without spending a lot of money [17].

Moreover, the cost of the models and required systems/equipment are also important in training [18]. Our objective in this technical report was two-fold. One, to construct a vessel model that demonstrates the shape of the vascular tree and resembles the handling/suturing properties of living tissue. And two, to construct that model affordably and suggest a cost-effective CBS training system to be used in conjunction with the training station.

Taken together, the material cost for an MCA model is less than $5. This price is substantially cheaper than the price of other effective models, and our model is far easier to replicate when compared to the one made by the University of Washington [19]. While the internal mold is dissolved in chloroform to acquire a hollow model, the external mold, however, can be used repeatedly. Due to the cost-effective and straightforward construction steps, we believe our MCA model can be widely used by residents and neurosurgeons throughout the world. Moreover, other models could be produced following our construction step. In other words, with minor changes, any disease morphology can be added to the model, or the vessel wall tissue characteristic can be altered according to age [20], patient, or disease. This would allow the model to become a valuable resource for a wide range of case-specific training. Moreover, the model can be integrated with current simulation systems to produce more holistic training.

Study limitations

Limitations were present in assessing the material characteristic of our MCA model in an objective way. We didn’t perform any mechanical tests such as stiffness test, uniaxial tensile strength test, or needle insertion deformation test to demonstrate the accuracy of the vessel wall tissue characteristics compared to real MCA. Also, we didn't get feedback from practicing cerebrovascular surgeons or trainers about the model's ability to replicate human cerebral vasculature. Another limitation was the lack of hemodynamic flow and thrombosis. However, such flow can be provided via basic pump-based infusion. And to compare with other training models, further studies measuring improvement in CBS skills are required. Moreover, our training system using the smartphone camera was limited to two dimensions, which is contrary to neurosurgical microscopes that provide three-dimensional viewing angles. Furthermore, measurements of the DICOM-based virtual model of MCA (n=1) were compared with the means of measurements of DICOM-based MCA models (n=8).

## Conclusions

In this technical note, we aimed to report how to construct a CBS training system involving the DICOM-based MCA model that demonstrates the shape of the vascular tree and resembles the handling/suturing properties of living tissue along with the training station. We believe that our DICOM-based MCA model may revolutionize current training methods by providing a realistic and widely available option for CBS training throughout the world with its desirable features of cost-effectiveness, and straightforward construction steps. Moreover, once the DICOM-based MCA model is used with our training station, it may offer an option for trainers to gain and maintain CBS skills without limitations of time, cost, and space.

## References

[1] Cheikh A, Yasuhiro Y, Kasinathan S, Kawase T, Takao T, Kato Y (2019). Superficial temporal artery: middle cerebral artery bypass, our series of 20 cases, surgical technique and indications with illustrative cases. Asian J Neurosurg.

[2] Lawton MT (2018). Seven bypasses: tenets and techniques for revascularization. New York.

[3] Winkler EA, Yue JK, Deng H (2019). National trends in cerebral bypass surgery in the United States, 2002-2014. Neurosurg Focus.

[4] Abla AA, Uschold T, Preul MC, Zabramski JM (2011). Comparative use of turkey and chicken wing brachial artery models for microvascular anastomosis training. J Neurosurg.

[5] Mokhtari P, Tayebi Meybodi A, Lawton MT, Payman A, Benet A (2017). Transfer of learning from practicing microvascular anastomosis on silastic tubes to rat abdominal aorta. World Neurosurg.

[6] Rubio RR, Shehata J, Kournoutas I (2019). Construction of neuroanatomical volumetric models using 3-dimensional scanning techniques: technical note and applications. World Neurosurg.

[7] Sarker MD, Naghieh S, Sharma NK, Chen X (2018). 3D biofabrication of vascular networks for tissue regeneration: a report on recent advances. J Pharm Anal.

[8] Aljohani W, Ullah MW, Zhang X, Yang G (2018). Bioprinting and its applications in tissue engineering and regenerative medicine. Int J Biol Macromol.

[9] Mackle EC, Shapey J, Maneas E (2020). Patient-specific polyvinyl alcohol phantom fabrication with ultrasound and X-ray contrast for brain tumor surgery planning. J Vis Exp.

[10] Tanriover N, Kawashima M, Rhoton AL Jr, Ulm AJ, Mericle RA (2003). Microsurgical anatomy of the early branches of the middle cerebral artery: morphometric analysis and classification with angiographic correlation. J Neurosurg.

[11] Roopavath UK, Kalaskar DM (2017). Introduction to 3D printing in medicine. ResearchGate.

[12] Song HG, Rumma RT, Ozaki CK, Edelman ER, Chen CS (2018). Vascular tissue engineering: progress, challenges, and clinical promise. Cell Stem Cell.

[13] Ridwan-Pramana A, Idema S, Te Slaa S, Verver F, Wolff J, Forouzanfar T, Peerdeman S (2019). Polymethyl methacrylate in patient-specific implants: description of a new three-dimension technique. J Craniofac Surg.

[14] Santangelo G, Mix D, Ghazi A, Stoner M, Vates GE, Stone JJ (2018). Development of a whole-task simulator for carotid endarterectomy. Oper Neurosurg (Hagerstown).

[15] Morikawa T, Yamashita M, Odaka M (2017). A step-by-step development of real-size chest model for simulation of thoracoscopic surgery. Interact Cardiovasc Thorac Surg.

[16] Scerrati A, Trovalusci F, Albanese A (2019). A workflow to generate physical 3D models of cerebral aneurysms applying open source freeware for CAD modeling and 3D printing. Interdiscip Neurosurg.

[17] Bedi MS, Bhavthankar TD, Girijala MR (2019). Lazy glass microsurgical trainer: a frugal solution for microsurgical training. World Neurosurg.

[18] Ganju A, Aoun SG, Daou MR (2013). The role of simulation in neurosurgical education: a survey of 99 United States neurosurgery program directors. World Neurosurg.

[19] Cikla U, Sahin B, Hanalioglu S, Ahmed AS, Niemann D, Baskaya MK (2018). A novel, low-cost, reusable, high-fidelity neurosurgical training simulator for cerebrovascular bypass surgery. J Neurosurg.

[20] Ghazi A, Campbell T, Melnyk R, Feng C, Andrusco A, Stone J, Erturk E (2017). Validation of a full-immersion simulation platform for percutaneous nephrolithotomy using three-dimensional printing technology. J Endourol.

